# Left Ventricular Pseudoaneurysm Possibly Associated with Rivaroxaban Therapy Disappeared after Cessation of Rivaroxaban: Case Report and Literature Review

**DOI:** 10.70352/scrj.cr.25-0513

**Published:** 2025-10-11

**Authors:** Kentaro Kiryu, Gembu Yamaura, Daichi Takagi, Takeshi Arai, Itaru Igarashi, Yuya Yamazaki, Wataru Igarashi, Hiroyuki Nakajima

**Affiliations:** Department of Cardiovascular Surgery, Akita University Graduate School of Medicine, Akita, Akita, Japan

**Keywords:** left ventricular vent tube, iatrogenic left ventricular pseudoaneurysm, anticoagulant drug, rivaroxaban

## Abstract

**INTRODUCTION:**

Although iatrogenic left ventricular pseudoaneurysm (LVPA) is rare, its treatment strategy needs consideration. We report a case in which postoperative initiation of rivaroxavan led to iatrogenic LVPA, which resolved spontaneously after drug cessation.

**CASE PRESENTATION:**

A 69-year-old man was diagnosed with a 76-mm saccular aortic arch aneurysm. He underwent urgent total aortic arch replacement with a frozen elephant trunk. A left ventricular apex injury due to intraoperative vent insertion was repaired, and postoperative CT revealed no problems. While hospitalized, he developed atrial fibrillation and started taking rivaroxavan. At 3 months postoperatively, an LVPA, 10 mm in diameter, developed at the intraoperative repair site. The LVPA resolved spontaneously following discontinuation of rivaroxaban, thereby avoiding the need for reoperation.

**CONCLUSIONS:**

This case illustrates the potential for anticoagulation-associated LVPA formation in surgically repaired myocardium and supports the consideration of conservative management in selected cases.

## Abbreviations

AF
: atrial fibrillation
CPB
: cardiopulmonary bypass
DOAC
: direct oral anticoagulant
FET
: frozen elephant trunk
LV
: left ventricular
LVAD
: left ventricular assist device
LVPA
: left ventricular pseudoaneurysm
TAFI
: thrombin-activatable fibrinolysis inhibitor
TAVI
: transcatheter aortic valve implantation
TTE
: transthoracic echocardiography

## INTRODUCTION

Intraoperative manipulation or catheterization resulting in cardiac injury can result in LVPA, which, depending on size and symptoms, requires a decision as to whether additional intervention or continued follow-up should be performed.

In this report, we describe a case in which the intraoperative repair of a heart apex injury resulted in LVPA due to postoperative initiation of rivaroxavan, which resolved spontaneously upon rivaroxavan cessation, thus avoiding additional intervention.

A literature review was also performed to verify the specificity of this case in relation to previous reports about rare iatrogenic LVPA cases.

## CASE PRESENTATION

A 69-year-old man was diagnosed with a 76-mm saccular aortic arch aneurysm on CT and underwent urgent total aortic arch replacement with a frozen elephant trunk. Preoperative blood test results were unremarkable. Electrocardiography showed sinus rhythm without significant ST-T changes. TTE demonstrated preserved ejection fraction (69%), no valvular disease, and focal wall motion asynergy. Coronary angiography revealed no significant stenosis.

A median sternotomy was performed under general anesthesia, and CPB was established using ascending aortic perfusion and bicaval drainage. The LV vent tube (Williams Vent Catheter 18Fr, 305 mm, SENCO MEDICAL INSTRUMENT, Tokyo, Japan) was inserted through the right superior pulmonary vein without resistance. Cardiac arrest was achieved with cardioplegia following aortic cross-clamping. The aortic arch was resected at Zone 0, and FET (J Graft FROZENIX, Japan Lifeline, Tokyo, Japan) was deployed under circulatory arrest and antegrade cerebral perfusion. Arch reconstruction was then completed using a four-branched graft (J Graft SHIELD, Japan Lifeline).

**Fig. 1:**
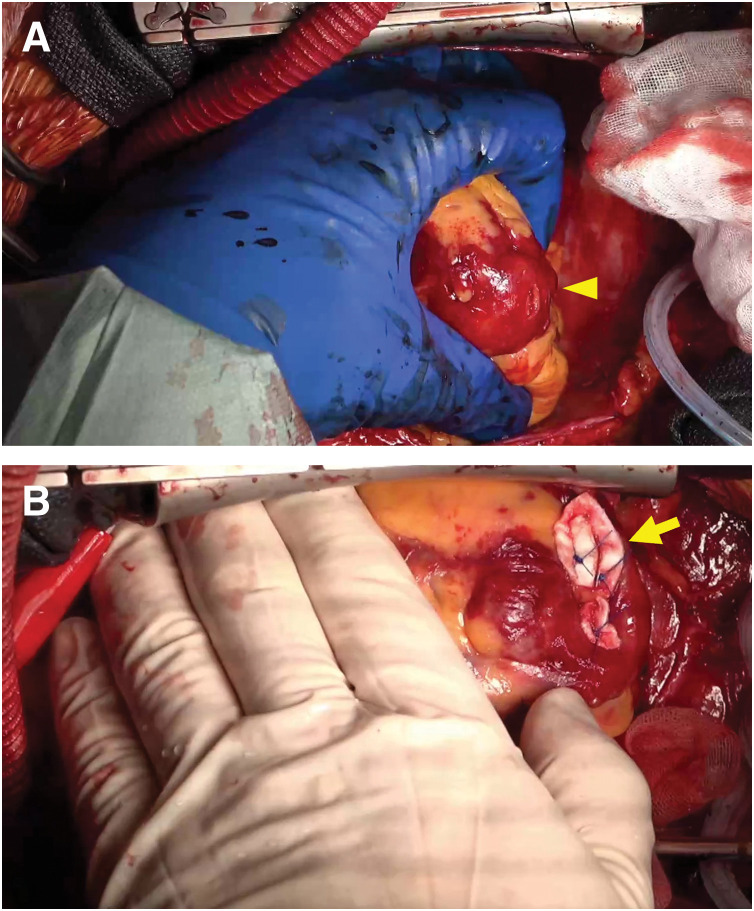
Intraoperative images. These show the left ventricular apical injury (**A**, arrowhead) and the repaired site following the felt sandwich technique (**B**, arrow).

During CPB weaning, active bleeding was observed from the LV apex, raising suspicion of vent tube-related injury (**Fig. 1A**). CPB was reinitiated, and hemostasis was achieved using a felt sandwich technique and mattress sutures (**Fig. 1B**).

**Fig. 2:**
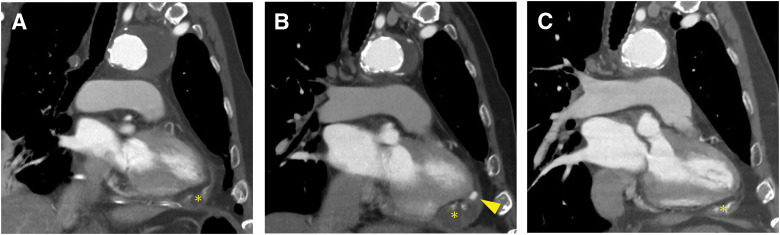
Comparison of perioperative CT. (**A**) CT on POD 8 shows only minimal pericardial fluid (asterisk) without significant extravasation. The apex repair site was thinned, with a wall thickness of 3 mm. (**B**) At 3 months postoperatively, a left ventricular pseudoaneurysm (arrowhead) with an associated fluid collection (asterisk) is evident. (**C**) Follow-up imaging at 3 months after discontinuation of rivaroxaban demonstrates resolution of the pseudoaneurysm and reduction of the fluid collection (asterisk). The thickness of the apical repair section was increased to 6 mm.

The patient was extubated on POD 2. On POD 3, he developed transient AF, which spontaneously reverted to sinus rhythm. CT on POD 8 showed no LVPA (**Fig. 2A**), and TTE on POD 13 demonstrated preserved cardiac function without valvular abnormalities. However, recurrent AF on POD 16 prompted initiation of DOAC therapy with rivaroxaban. The patient was discharged on POD 31.

Follow-up CT and TTE at 3 months postoperatively revealed a 10-mm LVPA at the apex, possibly associated with rivaroxaban use (**Fig. 2B**, **Supplementary Video 1**). Since CT and TTE had not been performed until this point, AF was detected for the first time at this stage. Given the absence of symptoms and maintenance of sinus rhythm, rivaroxaban was discontinued, and the patient was closely monitored. At 6 months postoperatively, CT confirmed complete resolution of the LVPA, with no recurrence observed at the 1-year follow-up (**Fig. 2C**, **Supplementary Video 2**).

### Literature review

We conducted a literature review of iatrogenic LVPA cases reported between January 2004 and December 2024 using PubMed. The search terms included combinations of: “iatrogenic LVPA,” “vent,” “TAVI,” “catheter,” “LVAD explant,” and “cannula.” The search was restricted to human adult cases and English-language publications. After screening abstracts and full texts, 15 case reports that met the inclusion criteria (iatrogenic cause, adult patients, and adequate clinical data) were extracted and summarized in **Table 1**.^1–15)^ Our case was added as the 16th entry for comparison. The results of the literature review are summarized in **Table 1**.

**Table 1:** Clinical and procedural summary of iatrogenic left ventricular pseudoaneurysm cases

No	Author Year	Patient primary disease	Intervention	Cause of LVPA	LVPA’s size Site Symptom	Anticoagulant drug	Intervention for LVPA	Outcome
1	Mekhail et al.^1)^ 2022	Female, 70 y.o TAAA	Two-stage aortic repair	Direct LV vent from the apex	24 × 19 mm Apex None	N/A	Percutaneous (Amplatzer)	Survived
2	Kasai et al.^2)^ 2023	Male, 82 y.o PVCs	Radiofrequency catheter ablation	Injury by the ablation catheter was suspected.	12 × 11 mm wall None	N/A	Conservative	Survived
3	Doost and Yong^3)^ 2021	Female, 90 y.o Aortic valve disease (no details given)	TA-TAVI	No LVPA until 1 year postoperatively, but at the same time, AF was observed, so the patient started taking warfarin. LVPA was noted at TTE 2 years after surgery (1 year after initiation of warfarin).	40 × 51 × 60 mm Apex None	Yes (warfarin)	Percutaneous (Amplatzer)	Survived
4	Vignati et al.^4)^ 2009	Male, 67 y.o IHD	Implantation of CRT device	Intraoperative left ventricular rupture in procedure for left ventricular epicardial lead implantation, closed with pericardial patch	60 × 70 mm Wall N/A	N/A	Percutaneous (Amplatzer)	Survived
5	Vidori et al.^5)^ 2025	Female, 87 y.o MR	TMVI	The development of LVPA was related to the use of a Safariextra small guidewire during the TMVI.	50 × 35 × 56 mm Apex Cardiac failure	N/A	Percutaneous (Amplatzer)	Survived
6	Patenè et al.^6)^ 2008	Male, 61 y.o Infective pleuro-pericarditis	Pericardiocentesis	Pericardiocentesis was interrupted due to the suspicion of left ventricularpuncture.	26 × 36 mm Apex Cough and fever	N/A	Surgery (bovine pericardial patch)	Survived
7	Moharana et al.^7)^ 2010	Female, 25 y.o Tuberculous pericardial effusion	Pericardiocentesis	Multiple pericardiocenteses were performed, but there was no complication.	90 × 80 mm Wall None	Post-LVPA’s treatment with warfarin	Surgery (Dacron patch)	Survived
8	Huang et al.^8)^ 2012	Female, 68 y.o TAAAD	Emergency repair (no details given)	Caused by vent tube insertion	Not mentioned Apex None	N/A	Surgery (running suture)	Survived
9	Czopak and Chaykovska^9)^ 2022	Male, 76 y.o Aortic arch aneurysm	TEVAR	Due to guidewire	60 mm Wall Cardiac failure	N/A	Percutaneous coil (after failed Amplatzer)	Survived
10	Yang et al.^10)^ 2012	Female, 61 y.o TAAAD	TEVAR	Due to guidewire	Not mentioned Apex Chest pain	N/A	Surgery (running suture)	Survived
11	Alkhouli et al.^11)^ 2017	Female, 71 y.o TR, constrictive pericarditis, IHD	Pericardiectomy, tricuspid valve ring repair, and CABG.	Caused by vent tube insertion	Not mentioned Apex None	N/A	Percutaneous (Amplatzer)	Survived
12	Angouras et al. ^12)^ 2020	Female, 72 y.o TAAAD	Hemiarch repair	Caused by vent tube insertion	20 mm Wall Not mentioned	N/A	Conservative	Survived
13	Kurdi et al.^13)^ 2024	Male, 46 y.o DCM, AR LVAD implant	LVAD wean and explant	Embolization of injury at the apical cannula extraction site with the Amplatzer device. However, a blood leak at the same site was observed.	Not mentioned Apex None	N/A	Surgery (running suture)	Survived
14	Montero-Cabezas et al.^14)^ 2021	Male, 80 y.o TAAAD	Hemiarch repair	Assumed to be an iatrogenic LVPA, but no details given.	Not mentioned Apex Cardiac failure	N/A	Percutaneous (Amplatzer)	Survived
15	Cappai et al.^15)^ 2016	Female, 76 y.o MR	TA-TMVI (Valve-in-valve)	Due to transapical approach	Not mentioned Apex None	N/A	Percutaneous (Amplatzer)	Survived
16	Present case	Male, 69 y.o Aortic arch aneurysm	TARFET Intraoperative apex injury due to vent tube insertion.	The LVPA was detected after the initiation of rivaroxaban.	10 mm Apex None	Yes (rivaroxaban)	Conservative (withdrawal of rivaroxaban)	Survived

AF, atrial fibrillation; AR, aortic valve regurgitation; CABG, coronary artery bypass grafting; CRT, cardiac resynchronization therapy; DCM, dilated cardiomyopathy; IHD, ischemic heart disease; LV, left ventricle; LVAD, left ventricular assist device; LVPA, left ventricular pseudoaneurysm; MR, mitral valve regurgitation; N/A, not applicable; PVC, premature ventricular contraction; TAAA, thoracoabdominal aortic aneurysm; TAAAD, type A acute aortic dissection; TARFET, total arch replacement with frozen elephant trunk; TA-TAVI, trans-apical transcatheter aortic valve implantation; TA-TMVI, trans-apical transcatheter mitral valve implantation; TEVAR, thoracic endovascular aortic repair; TR, tricuspid regurgitation; TTE, transthoracic echocardiography

### Patient profile and etiology

Among 16 adult cases of iatrogenic LVPA, the median age was 72 (range: 25–90) years, and 43.8% of patients were male.

Vent‑tube or trans‑apical access–related trauma accounted for 10/16 cases (62.5%), guidewire injury for 3/16 (18.8%), pericardiocentesis for 2/16 (12.5%), and epicardial lead implantation for 1/16 (6.3%).

### Morphology and symptoms

The pseudoaneurysm most commonly originated from the LV apex (14/16, 87.5%), with LV wall involvement in only 2 cases. Reported maximal diameters ranged from 10 to 90 mm. Half of the patients (8/16) were asymptomatic, while the remainder presented with chest pain or signs of heart failure.

### Role of anticoagulation

Only 3 patients had a history of anticoagulant use. In the case described by Moharana et al.,^7)^ warfarin was initiated after LVPA treatment, indicating a post-treatment introduction unlikely to be causally related to LVPA development. In the case described by Doost and Yong,^3)^ LVPA was identified 1 year after warfarin initiation; the pseudoaneurysm was large, requiring percutaneous intervention. By contrast, our present case is unique in that the pseudoaneurysm emerged shortly after initiating rivaroxaban and resolved following its discontinuation, a temporal sequence that has not been previously reported.

### Treatment and outcomes

Percutaneous plug closure was performed in 8/16 cases (50%), surgical repair in 5/16 cases (31.3%), and conservative management in 3/16 cases (18.8%). All patients survived the index admission. In the conservatively managed group, all LVPA diameters were ≤30 mm. Spontaneous resolution was observed only in the case reported by Angouras et al.^12)^ (vent‑tube-related LVPA, 20 mm) and in the present case of a 10‑mm rivaroxaban‑associated LVPA.

## DISCUSSION

Iatrogenic LVPA is a complication of catheterization,^2,4,9,10)^ pericardiocentesis,^6,7)^ injury due to vent tube insertion,^1,8,11,12)^ trans-apical transcatheter valve implantation,^3,5,15)^ and other procedures. Although symptomatic or large LVPAs (>30 mm) typically require surgical intervention, asymptomatic and smaller lesions (<30 mm) may be managed conservatively.^16)^

Our literature review identified 16 adult cases of iatrogenic LVPA, including the present case (**Table 1**). In most cases, LVPAs were managed surgically or with percutaneous intervention. Conservative management was reported in only 3 cases (Cases 2, 12, and 16), all of which involved LVPAs measuring ≤30 mm, consistent with the treatment cascade proposed by Prêtre et al.^16)^.

Only 2 cases (Cases 3 and 16) were associated with the initiation of anticoagulation therapy. In the case reported by Doost and Yong,^3)^ postoperative follow-up was initially uneventful; however, AF was later diagnosed, prompting the initiation of warfarin. One year after starting warfarin, an LVPA measuring 40 × 51 × 60 mm was detected, necessitating percutaneous intervention.

In our case, postoperative examination revealed no abnormalities at the intraoperative repair site. During hospitalization, the patient developed AF, for which rivaroxaban was initiated. Three months later, a 10-mm LVPA was detected. Given its small size, absence of symptoms, and the patient’s return to sinus rhythm, rivaroxaban was discontinued, and close follow-up was initiated. Spontaneous resolution of the LVPA was confirmed 3 months later.

This observation underscores the potential vulnerability of surgically repaired myocardium to hemorrhagic complications of anticoagulation. While interventional therapy is often the standard treatment for LVPA, this case highlights the importance of individualized evaluation and the potential for successful nonoperative management in appropriately selected patients.

Interestingly, in our case, LVPA did not develop immediately after surgery but emerged following the initiation of DOAC (rivaroxaban) therapy. This temporal association suggests that anticoagulation may have contributed to disruption at the site of the surgically repaired apex.

Recent studies have highlighted the differential effects of DOACs on fibrinolysis. Dabigatran, in particular, has a notable profibrinolytic effect by shortening clot lysis time through the inhibition of TAFI activation.^17)^ Rivaroxaban has also been shown to enhance fibrinolysis by enabling the tissue plasminogen activator cofactor function of FXaβ,^18)^ although its effect is less pronounced than dabigatran.

By contrast, apixaban and edoxaban appear to exert minimal or no profibrinolytic activity in comparable experimental models,^19)^ suggesting that their use may pose a lower risk for compromising the integrity of surgically repaired myocardial tissue.

Moreover, warfarin lacks any well-established profibrinolytic effects and may therefore represent a safer alternative in patients with recent myocardial repair. Although warfarin exerts its anticoagulant effects by reducing vitamin K-dependent coagulation factors, it is not known to directly enhance fibrinolysis. However, a study by Incampo et al.^20)^ reported that thrombi from warfarin-treated patients dissolved significantly faster than those from healthy controls (*P* = 0.001). The proposed mechanism involved hypoprothrombinemia, which leads to reduced activation of TAFI, which promoted fibrinolysis.

In our literature review, 1 case (Case 3) involved the development of LVPA following the initiation of warfarin, suggesting that indirect enhancement of fibrinolysis via TAFI suppression may have compromised the integrity of a surgically repaired myocardium. This finding underscores the importance of close postoperative monitoring and careful consideration of the necessity and duration of anticoagulation therapy when initiated after myocardial repair.

Taken together, these findings indicate that when postoperative anticoagulation is necessary following cardiac surgery involving myocardial repair, careful selection of anticoagulant agents is warranted.

Reports of spontaneous LVPA resolution following anticoagulation cessation are rare. In the present case, a previously repaired LV injury progressed to LVPA after initiation of DOAC therapy. The small size and asymptomatic presentation of the LVPA allowed for conservative management with anticoagulation withdrawal and close surveillance, resulting in complete resolution without further intervention. This case underscores the potential contribution of anticoagulation to LVPA formation and highlights the possibility of non-surgical management in selected patients.

Small (<30 mm) asymptomatic LVPAs may be managed conservatively with close follow-up. However, when postoperative anticoagulation is necessary, agents with minimal profibrinolytic activity, such as apixaban, edoxaban, or warfarin, may be preferable to dabigatran or rivaroxaban. Further prospective studies are required to validate this anticoagulant selection strategy.

## CONCLUSIONS

We report a rare case of LVPA that developed during rivaroxaban therapy and resolved after cessation, suggesting a potential association. In selected patients with small, asymptomatic LVPA, careful withdrawal of anticoagulation and close monitoring may obviate the need for surgical intervention. Furthermore, during the postoperative anticoagulation initiation period, careful consideration of fibrinolytic effects might be necessary when selecting medications to avoid rebleeding at the injury site or the formation of pseudoaneurysms.

## SUPPLEMENTARY MATERIALS

Supplementary Video 1 Transthoracic echocardiogram at 3 months postoperatively. It shows an echo-free space at the left ventricular apex, with visible inflow of blood into the pseudoaneurysm cavity.

Supplementary Video 2 Transthoracic echocardiography at 3 months after discontinuation of direct oral anticoagulant therapy. It demonstrats complete resolution of the echo-free space at the left ventricular apex.
